# Proton diffusion in the catalytic layer for high temperature polymer electrolyte fuel cells

**DOI:** 10.1039/c9ra06431a

**Published:** 2019-11-20

**Authors:** Marina Appel, Galin Borisov, Olaf Holderer, Marie-Sousai Appavou, Reiner Zorn, Werner Lehnert, Dieter Richter

**Affiliations:** Jülich Centre for Neutron Science at MLZ, Forschungszentrum Jülich GmbH Lichtenbergstr. 1 85747 Garching Germany o.holderer@fz-juelich.de; Institute of Energy and Climate Research, Forschungszentrum Jülich GmbH 52425 Jülich Germany; Acad. Evgeni Budevski, Institute of Electrochemistry and Energy Systems, Bulgarian Academy of Science 1113 Sofia Bulgaria; Jülich Centre for Neutron Science, Forschungszentrum Jülich GmbH 52425 Jülich Germany; RWTH Aachen University, Faculty of Mechanical Engineering 52062 Aachen Germany

## Abstract

The present study focuses on quasielastic neutron scattering (QENS) of the proton dynamics in phosphoric acid (PA) inside the catalytic layer of high-temperature polymer electrolyte fuel cells (HT-PEFCs). The nanosecond proton dynamics is investigated on the local length scale around operating temperatures (300 K–430 K) using neutron backscattering spectroscopy. We have investigated the catalyst doped with different amounts of PA in order to understand the distribution of PA inside the layer. Three approaches are considered for the description of proton dynamics: the random jump diffusion model, distribution of diffusion constants and, finally, the trap model. Due to adsorption of the PA on the Pt particles the diffusion of protons in the catalytic layer is different in comparison to the bulk acid. The proton dynamics in the catalytic layer can be described by the random jump diffusion with traps. This diffusion is significantly slower than the diffusion of free PA; this also results in a lower conductivity, which is estimated from the obtained diffusion constant.

## Abstract 2

The present study focuses on quasielastic neutron scattering (QENS) of the proton dynamics in phosphoric acid (PA) inside the catalytic layer of high-temperature polymer electrolyte fuel cells (HT-PEFCs).

## Graphical abstract

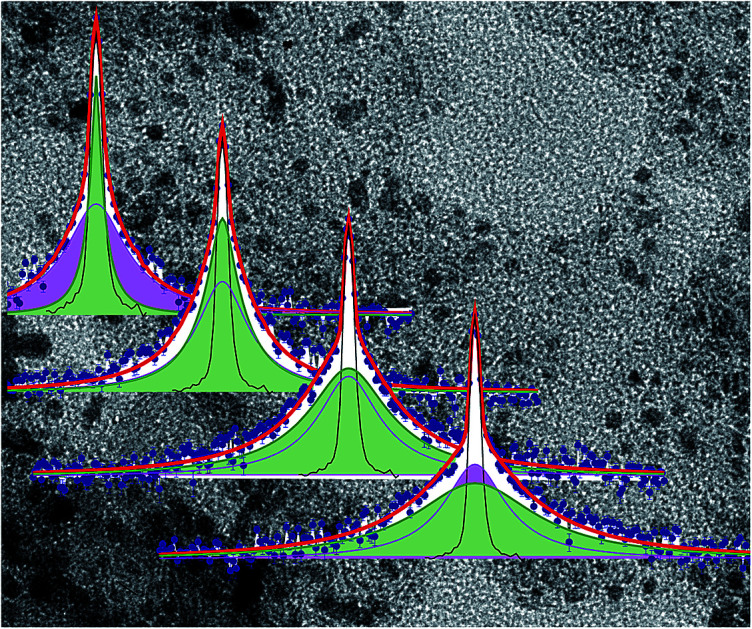

## 1. Introduction

Phosphoric acid-based polymer electrolyte fuel cells operating at elevated temperatures (160 °C–180 °C) attract increasing attention for different applications in the kW power range.^1–5^ The advantages of this type of proton exchange fuel cells include low sensitivity to CO contamination,^6^ no need for complicated water management and potentially high proton conductivity.^7,8^ The conductivity of such cells is directly proportional to the amount of phosphoric acid (PA), which has the highest intrinsic proton conductivity of any known substance.^9^ The dependence of the HT-PEFC performance on different operating parameters has been studied with operating stacks on a more macroscopic length scale.^10–13^ However, the conductivity of PA at the fundamental level is not yet fully understood. Recently, the mechanism of the proton conduction of PA has been described using *ab initio* molecular dynamics simulations.^14^ Neutron scattering has been employed to study PA.^15^ The experimental study of the proton dynamics of anhydrous PA on the local scale has been lately reported and compared with a random jump diffusion model.^16^ The situation with studies on proton conductivity inside the elements of the fuel cell is even more complicated. There is an obvious lack of information about proton dynamics inside the cells. It has been observed that in spite of high conductivity of the PA itself, the conductivity of the HT-PEFC is lower than expected. Due to the complex structure of the membrane electrode assembly (MEA) – the central part of the fuel cells – there are many factors which can influence the proton transport and cause losses of the cell performance. The understanding of the underlying physical processes of proton transport of PA in the MEA can help to solve problems in the proton conductivity and advance the design of HT-PEFC towards higher performance.

The MEA typically consists of a polybenzimidazole-type (PBI) membrane doped with PA and sandwiched between two gas diffusion electrodes.^17,18^ The electrode is the catalytic layer containing a carbon-supported Pt catalyst painted on the gas diffusion layer. Since most of the PA is concentrated in the membrane^19^ past research was mostly focused on proton transport in the membrane and not in the catalytic layer. The proton transport in the PBI membrane has been described theoretically^20,21^ using molecular dynamics simulation as well as experimentally using neutron scattering.^22^ However, the interaction of the PA with the catalytic layer can also contribute to the conductivity of the MEA. Since the catalytic layer is in direct contact with the PBI membrane doped with PA it also contains some amount of acid, which is needed to provide good proton conductivity in the electrode. On the other hand, too high acid loadings of the electrodes lead to a significant decrease in performance.^23^ Neutron scattering is an extremely powerful technique for studying the structure^24,25^ and proton diffusion by quasielastic neutron scattering^26^ in complex environments^27^ due to its unique sensitivity to hydrogen and the possibility of contrast variation with partly deuterated materials.^28^

Information about proton dynamics of PA in the catalytic layer is completely missing nowadays. In our previous work^29^ it has been briefly mentioned that the structure of the catalytic layer influences the proton dynamics of the PA.

In this paper we report on experiments addressing the dynamics of PA in the catalytic layer using high energy resolution neutron backscattering spectroscopy. Quasielastic neutron scattering (QENS) experiments directly probe the nanosecond proton dynamics in a broad *Q* and temperature range. Two identical electrodes loaded with different amounts of PA are analyzed and compared. In Section 2 we first describe the samples and methods, which are later used in the discussion in Section 3.

## 2. Experiments

### 2.1. Sample preparation

Gas diffusion electrodes were prepared from a catalytic powder consisting of Pt nanoparticles supported on carbon black with a metal weight fraction of 20% (HISPEC 2000). The catalytic powders were mixed with water and solvent (propan-1-ol : propan-2-ol (1 : 1)) using an ultrasonic finger. PTFE (Dyneon TF5032Z, 24%) was added to the prepared mixture. The obtained inks were coated onto a commercially available non-woven gas diffusion layer (GDL) with microporous layer (Freudenberg H2315C2) by an automated doctor blade technique and then dried in air over night.

In the next step the gas diffusion electrodes were doped with 85% phosphoric acid (H_3_PO_4_). The phosphoric acid was mixed with deuterated ethanol (C_2_D_5_OD) in a ratio 1 : 4 under inert atmosphere in order to control humidity of the sample and then dried in vacuum over several hours. Two samples with a final amount of acid of 100 μl (6.3 μl cm^−2^) and 60 μl (3.8 μl cm^−2^) have been prepared. Afterwards, the electrodes were sealed in specially designed Al sample containers. A thin PFA foil (thickness 0.25 μm) was used around the electrode to protect the Al sample container from corrosion. Before the experiment all samples were heated up to 180 °C for several hours and then cooled down to room temperature. The electrode layers with the two different doping levels have been prepared in the same way, the long time in the vacuum chamber removes excess water in the same way, such that both doping levels can be directly compared.

Samples were characterized with a transmission electron microscope JEM 2200 FS EFTEM instrument (JEOL, Tokyo, Japan) with zero-loss energy filtering. The sample preparation for the TEM images has been described in ref. 29.

### 2.2. Neutron backscattering spectroscopy

The dynamics of the PA was studied with QENS on the backscattering spectrometer SPHERES at the Heinz Maier-Leibnitz Zentrum (Garching), which provides access to dynamics on a time scale from a few hundred picoseconds up to a few nanoseconds, corresponding to an energy resolution of 0.66 μeV and a dynamical range of −31 μeV to 30 μeV, which can be achieved by moving the Si(111) monochromator on a Doppler drive. The detailed layout of the spectrometer can be found in literature.^30,31^

Both samples have been measured at several temperatures within the range of 300 K–430 K using the full *Q* range from 0.2 to 1.7 Å^−1^. The resolution function was obtained from a measurement at 3 K. At this temperature the scattering is considered to be purely elastic on the observation time scale.

Due to the large incoherent cross section of protons, neutron scattering is very sensitive to their motions. Proton dynamics in hydrogen-containing materials can be probed by incoherently scattered neutrons, for example using QENS. A detailed description of the basic principles of QENS can be found in literature;^32^ here only the necessary relations will be given.

The intensity of incoherently scattered neutrons is proportional to the incoherent scattering law *S*(*Q*,*ω*) which is the Fourier transform of the van Hove self-correlation function *G*_s_(*r*,*t*):1
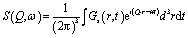
where *Q* and *ω* are related to momentum and energy transfer respectively.^33^ The function *G*_s_(*r*,*t*) describes the specific motion of the atoms and thus plays in fact the role of a model function, which then has to be Fourier transformed in order to obtain the experimentally measured *S*(*Q*,*ω*). Generally, the experimentally measured line broadening can be written in the following way

2 *S*(*Q*,*ω*) = *R*(*Q*,*ω*) ⊗ (*A*_1_(*Q*)*δ*(*ω*) + *A*_2_(*Q*)*L*(*Q*,*ω*)) + *bkg*

where *R*(*Q*,*ω*) is resolution function of the instrument, the first component in the brackets is an elastic contribution that may result either from immobile protons and/or from protons performing spatially restricted motion. Then this component is the Fourier transformed of the distribution of the proton at infinite times and is called elastic incoherent structure factor (EISF). The elastic part is described with a delta-function *δ*(*ω*) and the corresponding amplitude *A*_1_(*Q*). The second component is the QENS contribution which is depicted by spectral function *L*(*Q*,*ω*) with the amplitude *A*_2_(*Q*). The spectral function is defined by the chosen model of the microscopic motion. In the simplest model-free approach, *L*(*Q*,*ω*) can be chosen as a Lorentzian function with a characteristic width (half-width at half maximum *Γ*).

In the present work we consider three possible models for the proton diffusion such as random jump diffusion, the jump diffusion model with distribution of the diffusion constants and finally, the trap model. The random jump diffusion^34^ model describes the scattering from particles which oscillate for the time *τ*, the so-called residence time, and then jump to a distance *l*, defined as a jump length. In this case the spectral function *L*(*Q*,*ω*) is the Lorentzian line with width depending on *Q* in following way3
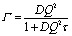
where *D* is the diffusion coefficient. The model approaches the standard continuous diffusion at low *Q*: *Γ* = *DQ*^2^ and at high *Q* the jump rate 1/*τ* defines the width of the line. The jump length can be obtained from *D* and *τ* as 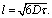 This model describes well the diffusion of bulk phosphoric acid.^16^

Since the catalytic layer has a multiporous structure with pore size from few nm to few hundreds nm the proton diffusion can be affected by the structural differences within the sample. Thus, instead of a uniform diffusion one would rather expect a distribution of diffusion constants. Strictly speaking, in case of the jump diffusion the jump length is a distributed parameter, since the jump length is sensitive to the structural constraints. However, for the simplicity of the analysis the distribution can be transferred to the diffusion coefficient due to the simple dependence between *l* and *D*. Thus, *D* can be distributed according log-normal distribution function:4
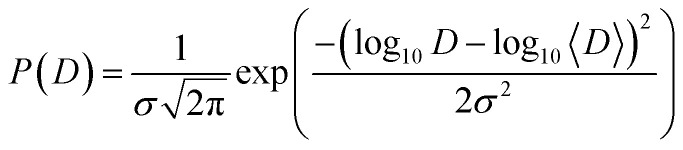


Then the spectral function *L*(*Q*,*ω*) is basically the integral over many Lorentzian lines with widths defined according eqn 3, where 〈*D*〉 is the geometric mean diffusion coefficient, and *σ* is the width of distribution.

The trap model^35^ is based on the jump diffusion model with assumption that protons can be trapped for some time in the structure of the catalytic layer and then released again. The traps are characterized by the mean trapping time *τ*_0_, which the protons spend in the trap. After escaping the trap, the proton diffuses, as it would without traps until it is trapped again. The mean time between trapping events is defined as *τ*_1_. Then the function *L*(*Q*,*ω*) has two components
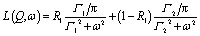
where the individual widths of both Lorentzian lines are defined as5
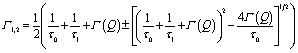
with the spectral weight6
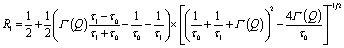


## 3. Experimental results and discussion

First of all, the obtained QENS spectra of PA in the catalytic layer were normalized to vanadium and direct scattering. Due to the quite low scattering signal the dry electrode was not directly subtracted from the data. Instead the QENS spectra of the dry electrode have been measured at the same temperatures as the doped electrodes and analyzed using eqn (2). It should be noted that scattering from the dry catalyst has a small QENS contribution, coming most probably from the PTFE contained in the electrodes. This QENS can be described with a Lorentzian function. The obtained parameters *i.e.* the width of the Lorentzian line and its amplitude have been included in the final fit function for the doped electrodes as an additional constant contribution.

**Fig. 1:**
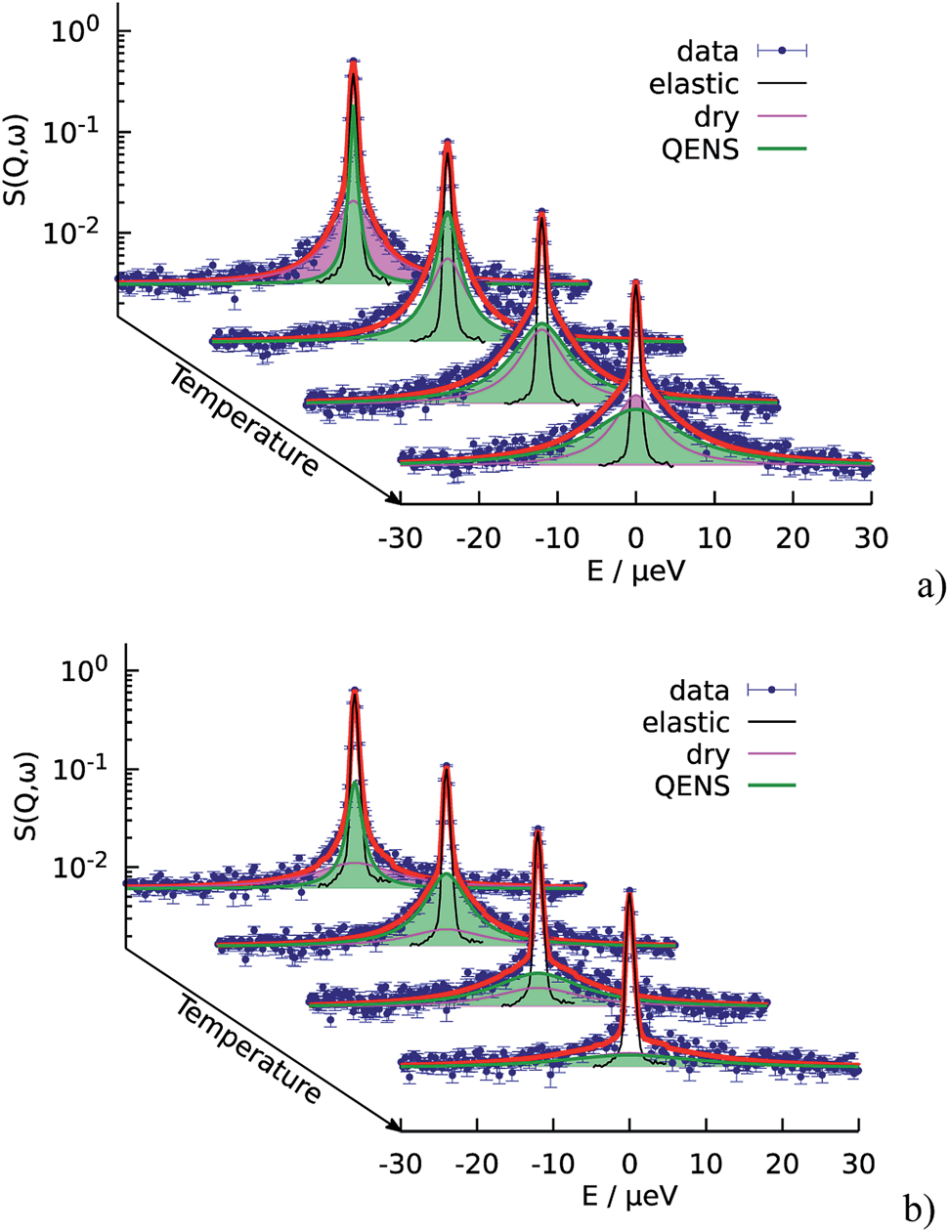
Temperature evolution of the QENS spectra of the catalytic layers doped with 100 μl of PA. The fit function according to the random jump diffusion model is shown in red. Temperatures from back to front: 300 K, 340 K, 380 K, and 430 K. (a) *Q* = 0.6 Å^−1^ (b) *Q* = 1.66 Å^−1^.

**Fig. 2:**
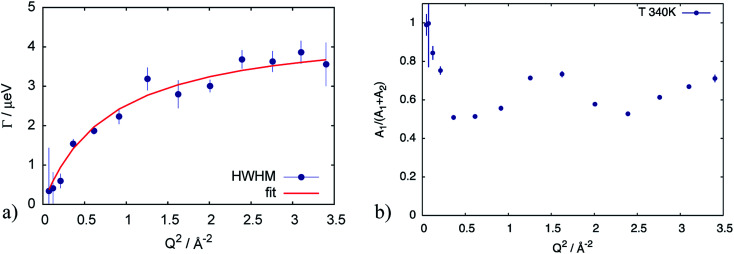
(a) HWHM of the Lorentzian line describing the QENS component of the spectrum at *T* = 340 K for the catalytic layer doped with 100 μl of PA. Red line corresponds to the fit according to eqn (3). (b) elastic contribution *A*_1_ normalized to the sum of contributions (*A*_1_ + *A*_2_) in eqn (2), reflecting the structure of the sample with a small angle scattering contribution from at low *Q*, and a broad peak presumably from the amorphous carbon support.

The QENS spectra of the catalytic layer doped with 100 μl of PA are shown in Fig. 1. One can observe the broadening of the peak with temperature. We can see that the QENS contribution develops from temperature to temperature: from small wings of elastic line at 300 K to well pronounced peaks at the high temperatures. Comparing two plots in Fig. 1 one can also observe the evolution of the spectra with *Q*. For example, at *T* = 430 K the *Q* = 0.6 Å^−1^ spectrum exhibits a clear QENS contribution while at *Q* = 1.66 Å^−1^ this contribution becomes too wide to be visible in the experimental window. As first step, the spectra have been fitted using one Lorentzian line convoluted with the resolution function; the resulting *Q*-dependence of the HWHM of the Lorentzian line is shown in Fig. 2. At small *Q* there is a linear dependence of the width with *Q*^2^ corresponding to continuous diffusion, however, at large *Q* the width deviates from the linear law and approaches a plateau. The red line in Fig. 2a shows a fit with the random jump diffusion model (eqn (3)), which follows well the experimental points. The resulting parameters are displayed in Table 1. The elastic contribution *A*_1_/(*A*_1_ + *A*_2_) is shown in Fig. 2b and reflects the structure of the electrode layer with a peak at *Q*^2^ = 1.5 Å^−2^ arising presumably from the amorphous carbon support and a small angle scattering contribution at low *Q*. The elastic contribution was temperature independent within the precision of the measurement.

**Table 1:** Fit parameters obtained with simple jump diffusion and with distribution of the diffusion constant

Doping level	*T*, K	*D*, 10^−7^ cm^2^ s^−1^	*Σ*	*τ*, 10^−2^ ns	*l*, Å	E_a_^*D*^, meV
**Simple jump diffusion**						
High	300	1.4 ± 0.4	—	70 ± 10	2.5 ± 0.4	330 ± 41
	340	6.0 ± 0.5	—	22 ± 1	2.8 ± 0.1	
	380	22 ± 2	—	9.4 ± 0.7	3.5 ± 0.2	
	410	44 ± 6	—	8.7 ± 0.7	4.8 ± 0.4	
	430	37 ± 6	—	5.4 ± 0.8	3.5 ± 0.4	
						
**Jump diffusion with distribution of diffusion constant**						
Low	300	0.6 ± 0.3	0.4	43 ± 17	1.3 ± 0.4	447 ± 38
	340	2.4 ± 0.2	0.7	7 ± 1	1.0 ± 0.1	
	380	16 ± 1	1.0	4.3 ± 0.9	2.0 ± 0.2	
	410	31 ± 5	1.3	5.0 ± 0.8	3.1 ± 0.3	
	430	43 ± 9	1.3	4 ± 1	3.2 ± 0.6	

**Fig. 3:**
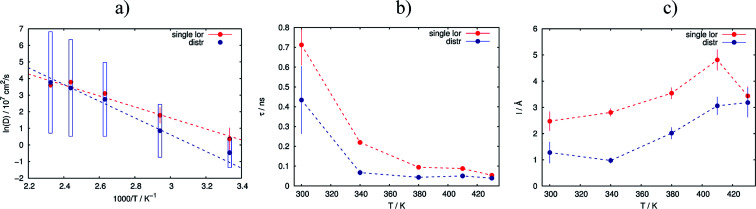
Variation of the obtained fit parameters with temperature for the simple random jump diffusion model (red lines) and the jump diffusion with distribution of the diffusion constants (blue lines). (a) Arrhenius plot of diffusion constants with fit; (b) residence time; (c) jump length.

As the next level of the analysis we take the jump diffusion as a model for the proton transport in the catalytic layer and implement eqn (3) directly into the Lorentzian of eqn (2) for *S*(*Q*,*ω*). Each temperature has been fitted independently. The resulting fit is shown in Fig. 1 as red line. QENS contributions arising due to proton motions of the PA as well as from the dry catalyst are also shown in the plot. The obtained fit parameters *D*, *τ* and *l* are summarized as a function of temperature in Fig. 3. One can observe that the diffusion coefficient follows an Arrhenius law, and the residence time, as expected, decreases with temperature. The high value of *τ* at 300 K can be explained by the weak scattering signal of PA. The broadening of the elastic line at this temperature is determined mostly by the scattering of the dry catalytic layer. Therefore only at higher *Q* the contribution of the PA plays a significant role. This is also clearly visible in Fig. 1. The weak QENS contribution of the PA together with relatively large signal from the dry electrode leads to a high uncertainty and instability of the fit routine. A similar argument holds for the highest measured temperature of 430 K where the QENS signal of PA is getting too broad, and at high *Q* partially moves out of the dynamical window of the spectrometer; the *Q* range where the jump diffusion model can be applied is significantly narrowed. Nevertheless, the jump diffusion model provides quite reasonable results on the investigated temperature range. However, for the lower doping level we could not obtain a systematic variation of the fit parameters, which might suggest that the jump diffusion model is not a complete description of the system.

**Fig. 4:**
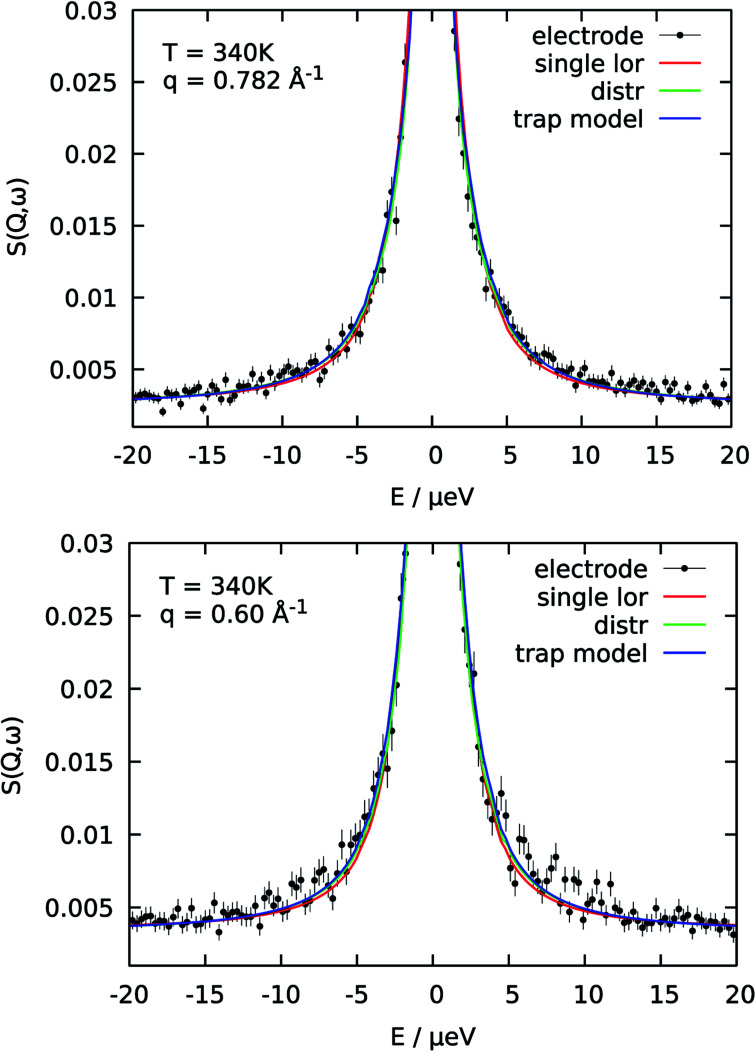
Comparison of three different model functions for the QENS spectra of catalytic layer doped with 100 μl of PA for two different *Q*-values. For a single *S*(*Q*,*ω*) all models fit the data well. Using common parameters for all PA concentrations and temperatures is then only possible with the trap model.

The reason for the deviation of the proton diffusion in the catalytic layer from the random jump model could be the heterogeneous structure of the layer itself. Since the layer is a porous material diffusion with slightly different parameters can occur in different regions within the sample. To account for this heterogeneity in the diffusion we include the distribution of the diffusion coefficients into the jump diffusion model according to eqn (4). Thus, instead of one diffusion coefficient *D* we obtain the mean geometric diffusion coefficient 〈*D*〉 with a certain width of the distribution *σ*. The difference of the quality of the fit between the simple jump diffusion model and the model with included distribution is shown in Fig. 4.

As we can see, the distributed model accounts better for the wings of the scattering curve. The resulting parameters of the fit are shown in Fig. 3 in blue. Again 〈*D*〉 follows an Arrhenius behavior. At high temperatures the absolute value of the mean diffusion coefficient is similar to the one obtained in the case of the simple jump diffusion, and there is a deviation between two models at low temperatures. The width of the distribution *σ* increases with temperature, and at high *T* the diffusion coefficient *D* is even distributed over multiple orders of magnitude *D*. The large sigma suggests that there are some broad components with respect to 〈*D*〉, and the overall diffusion process is very heterogeneous. Another explanation can be that there are simply two components with significantly different widths of the Lorentzian lines, which will also lead to an apparent large *σ* in the distributed model. On the other hand, such a broad *σ* leads to instability of the fitting routine due to simultaneous fitting of *σ* and the amplitudes *A*_1_ and *A*_2_ (see eqn (2)), which are increasingly correlated for large sigma. When the distribution is very broad many Lorentzian lines lie in the range of the elastic line and *σ* starts to correlate with the elastic amplitude. In this case the fitting model cannot really account the very narrow components properly, which of course can result to some systematic errors in other parameters, for example the constant value of residence time *τ* (Fig. 3b), which in principle should decrease with the temperature.

The jump length *l* obtained from *D* and *τ* increases with temperature from 1 Å to 3 Å, which is significantly lower than the jump length obtained from the simple jump diffusion model.

Analysis of the sample with lower doping level again does not give a consistent and systematic variation of the fit parameters. This also would speak against the distributed jump diffusion model for the proton dynamics in the catalytic layer.

As was already mentioned above, a the QENS spectra may also be parametrized in terms of a two components model, containing one narrow and one broad QENS line. One such model is the trap model, describing the diffusion processes in a system with traps. For the diffusion in the catalytic layer this model has a physical basis – adsorption of the hydrogen on the surface of the Pt particles.^36^ Due to the interaction of PA with Pt particles the protons can be partially immobilized and will not participate in the diffusion for some time. The quality of fitting with the model is shown in Fig. 4 in blue. The trap model better describes the experimental spectra than the simple jump diffusion model or model with distribution of the diffusion coefficient. However, the model introduces two more fit parameters and some assumptions have to be done.

First of all, the model contains two parameters *D* and *τ*, which describe diffusion of the free PA in terms of random jump diffusion. These parameters do not depend on the sample structure and the doping level of the catalytic layer. Thus, *D* and *τ* can be kept as global parameters for both the highly and the low doped samples. The two new parameters *τ*_0_ and *τ*_1_ take into account the traps as explained in Section 2.2. These parameters are individual for each sample and can vary depending on the doping level. In order to reduce the amount of free fit parameters we introduce an analytical temperature dependence of *D* and *τ*_0_. This allows fitting all temperatures at the same time. As we have seen above the diffusion coefficient follows an Arrhenius law; instead of fitting a diffusion constant per temperature we can write it in the Arrhenius form:7
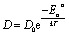
where *E*_a_^*D*^ is the activation energy of the diffusion process and *k* is the Boltzmann constant. Strictly speaking *D* should not follow an Arrhenius law, but a Vogel–Fulcher–Tammann (VFT) law.^37–40^ However, the limited amount of temperature points in the quite narrow temperature range can be well described with an Arrhenius law. The trapped protons will escape the trap after the time *τ*_0_, and as the escape of the protons is also a thermally activated process, the escape rate 1/*τ*_0_ can be described by an Arrhenius law as well:8
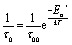
with activation energy *E*_a_^*τ*^. Since the structure of the both samples does not change with temperature during the experiment we assume that the mean-squared distance between trapping events *s*^2^ = 6*Dτ*_1_ is temperature independent.

**Fig. 5:**
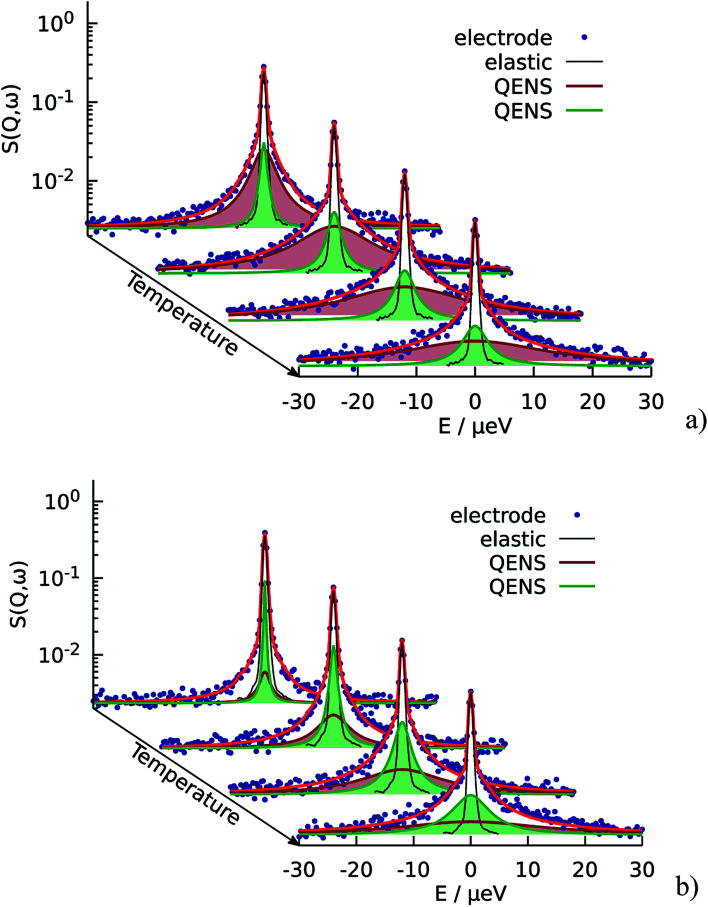
QENS spectra of the catalytic layers doped with (a) 100 μl and (b) 60 μl of PA. Red lines correspond to the fit according to the trap model. Two QENS components are highlighted in green and brown. The QENS contribution from dry catalyst is not shown here, but it is taken into account during the fit.

**Fig. 6:**
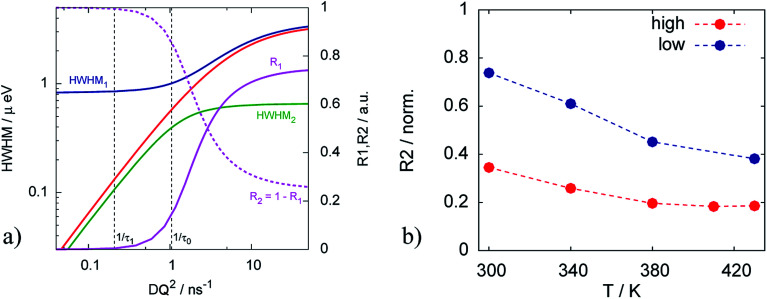
(a) Widths of the two components *Γ*_1_ and *Γ*_2_ of the trap model and their spectral weights at *T* = 340 K. Red line is the width *Γ* of the free PA. (b) Amount of the trapped protons (estimated from the *R*_2_ at the highest *Q*) in the catalytic layer for different doping levels.

The resulting fits are shown in Fig. 5a where two components of the model are highlighted. One can observe that the broad component dominates over the investigated temperature range, while the narrow component adds a smaller contribution to the fit function. Fig. 6a illustrates the behavior of the two components, namely their widths and spectral weights as a function of *Q*^2^ obtained from the fit at *T* = 340 K. The vertical black dashed lines show 1/*τ*_0_ and 1/*τ*_1_; for the obtained fit parameters these values lie below the first measured *Q* value (for the first measured point *DQ*^2^ = 1.7 ns^−1^). In the experimentally measured *Q* range both spectral components contribute to the total spectrum. The width of the broad component monotonously increases with *Q*; the shape of *Γ*_1_ is similar to the shape of *Γ* of the free PA (red line in Fig. 6a). The spectral weight of this component increases with *Q*. At small *Q* this component has a weak contribution to the spectrum, and it dominates at large *Q*. The width of the narrow component is nearly constant almost over the entire *Q*-range except for the few smallest values. The weight of the narrow component at high *Q* is proportional to the amount of the trapped protons, which is shown in Fig. 6b as a function of temperature. The amount of trapped protons is reduced by a factor of 2 between 300 K and 430 K; this happens due to the higher activation energy for the escape rate than for the diffusion. The parameters of the trap model can be found in Table 2.

**Table 2:** Fit parameters obtained with trap model for high and low doping levels of the catalytic layer

Doping level	*T*, K	*D*, 10^−7^ cm^2^ s^−1^	*τ*, 10^−2^ ns	*l*, Å	*τ*_0_, ns	*τ*_1_, ns	*D*_eff_, 10^−7^ cm^2^ s^−1^	〈*s*^2^〉, Å^2^	*E*_a_^*D*^, meV	*E*_a_^*D*_eff_^, meV	*E*_a_^*τ*^, meV
High	300	7.0 ± 0.1	65 ± 3	5.2 ± 0.2	2 ± 1	9.8 ± 0.4	5.8 ± 0.7	413 ± 39	155.4 ± 1.7	157 ± 1	165 ± 18
	340	14.3 ± 0.3	19 ± 1	4.0 ± 0.2	1.0 ± 0.6	4.8 ± 0.3	12 ± 1				
	380	24.9 ± 0.9	4.0 ± 0.2	2.4 ± 0.1	0.5 ± 0.3	2.8 ± 0.2	21 ± 2				
	410	35 ± 2	1.9 ± 0.1	2.0 ± 0.1	0.4 ± 0.2	1.9 ± 0.2	30 ± 2				
	430	43 ± 7	1.8 ± 0.1	2.2 ± 0.2	0.3 ± 0.1	1.6 ± 0.2	37 ± 3				
Low	300	7.0 ± 0.1	65 ± 3	5.2 ± 0.2	3 ± 2	2.7 ± 0.2	3 ± 1	115 ± 12	155.4 ± 1.7	170.0 ± 0.5	197 ± 1
	340	14.3 ± 0.3	19 ± 1	4.0 ± 0.2	1.0 ± 0.5	1.3 ± 0.2	8 ± 2				
	380	24.9 ± 0.9	4.0 ± 0.2	2.4 ± 0.1	0.4 ± 0.2	0.8 ± 0.1	16 ± 3				
	430	43 ± 7	1.8 ± 0.1	2.2 ± 0.2	0.18 ± 0.08	0.44 ± 0.08	31 ± 4				

The situation is different for the sample with lower doping level. As we can see on Fig. 5b the narrow component is dominant in this case. It has higher contribution at all *Q* and temperatures, which means that the relative amount of trapped protons is significantly higher than for the sample with the higher doping level, which is also illustrated in Fig. 6b Thus, the trapping sites are attractive for the PA and at lower doping the empty trapping sites are filled first and the remaining relative amount of free PA is lower than in the case of high doping.

The mean-squared distance between trapping events 〈*s*^2^〉 obtained from the fit is significantly different for different doping levels: 413 ± 39 Å^2^ for the sample with high doping level and 115 ± 12 Å^2^ for the low doping level. Since the traps are assumed to be a property of the structure of the catalytic layer, and this structure is identical for both samples, there should be no difference in 〈*s*^2^〉. The reason of this discrepancy could be the high amount of protons which are never trapped in the sample with the higher doping level. In other words, this sample contains some amount of free PA and an additional contribution describing the diffusion of the free PA should be included in the fit function. In the sample with the low doping level this additional contribution is negligible since the amount of the PA is not enough to completely fill the pores of the catalytic layer. Re-fitting the spectra with one more component described by *D* and *τ* (see eqn (2)) gives the fraction of free PA, which depends on temperature and increases from 0.5 to 0.65 with *T*. Table 3 shows the change of the temperature independent parameters for the high doping level, which are within the errorbars identical to the low doping level parameters. Thus, diffusion of only half the protons can be described by the trap model (with the diffusion coefficient denoted *D*_eff_ to distinguish it from the simple jump diffusion coefficient *D*), while the other half follows simple jump diffusion. This is also the explanation why the sample with high doping level could be satisfactory described with only the jump diffusion model, while the fit did not work for the sample with lower doping level where the contribution of the trapped protons is much higher.

**Table 3:** Final fit parameters of the trap model including the fraction of free PA

Doping level	〈*s*^2^〉, Å^2^	*E*_a_^*D*^, meV	*E*_a_^*D*_eff_^, meV	*E*_a_^*τ*^, meV
High	115 ± 12	155.4 ± 1.2	168.0 ± 1.0	204 ± 2
Low	115 ± 12	155.4 ± 1.2	170.9 ± 0.5	197 ± 1

The fraction of free PA in the sample with the low doping level was kept fixed to 0 due to simplicity. A fit where this parameter was free suggests that for low doping the fraction of free PA is less than 5% at all temperatures.

**Fig. 7:**
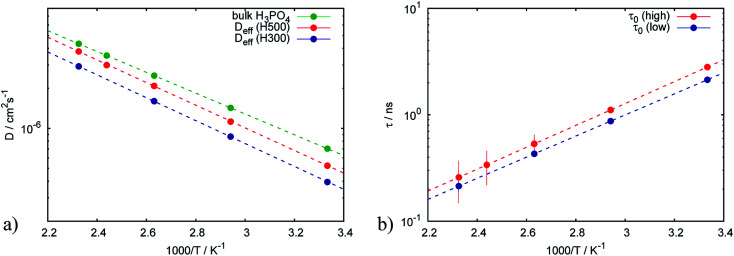
Arrhenius plots of (a) effective diffusion coefficients and (b) trapping time obtained from the trap model for catalytic layers with different doping levels.

The Arrhenius behavior of the diffusion coefficient *D* and the times *τ*_0_ and *τ*_1_ are shown in Fig. 7. The green points on Fig. 7a show the free PA, described by the diffusion coefficient *D*, red and blue lines represent effective diffusion coefficients for high and low doping levels respectively. Taking into account the fraction of free PA, the activation energies for both samples do not differ much: 170.9 ± 0.5 meV and 168.0 ± 1.0 meV for low and high doping levels correspondingly. Knowing the diffusion coefficients we can estimate the conductivity *σ* of PA in the electrodes using the Nernst–Einstein equation:^41^

9 *σ* = *N*_v_*De*^2^/*kT*

where *N*_v_ is the charge density which is estimated to *N*_v_ = 3 × 10^28^ m^−3^ for PA,^22^*e* is the elementary charge and *k* is the Boltzmann constant. The obtained conductivity for the free PA is consistent with known values^42^ and equals to *σ* = 0.56 S cm^−1^ at *T* = 430 K. Due to the trapping of the protons the conductivity of the PA in the catalytic layer is accordingly lower than in the bulk and equals to 0.49 S cm^−1^ for high doping level and 0.38 S cm^−1^ for the low one.

Fig. 7b shows that the trapping time in both cases is very similar, which indicates that the nature of traps is the same and defined by the structure of the catalytic layer and does not depend on the amount of acid.

It has to be emphasized that the two samples with different doping levels have been prepared in the same way, and can thus be compared directly. The sample storage in the vacuum chamber guarantees that the phosphoric acid in both samples has the same water contents, which depends on the partial water pressure and temperature during storage.^43^ The phosphoric acid had sufficient time for equilibrating with these conditions. Storage under vacuum will lead to a concentration of the PA, since the partial pressure of the H_3_PO_4_ is negligible and only water will evaporate, *i.e.* the concentration is higher than 85%. The “bulk” PA (or free PA) diffusion constant in Fig. 7 is the diffusion constant of the fast component of the QENS spectra present in the electrode. The conductivity at 430 K according to ref. 43 is varying only by approx. ±0.05 around its value of 0.56 S cm^−1^ depending on the exact PA composition. We think therefore it is justified to assign this component to free PA in the electrode layer. More details of the chemical and thermodynamic properties of PA and the different PA species depending on temperature, pressure and water contents can be found in ref. 43.

The decreasing jump length with temperature is somewhat intriguing. A hypothesis might be that in the energy landscape the protons find more easily a nearby place with higher temperature, thus reducing the jump length. In other words, at higher temperatures more fluctuations increase the probability of a free nearby position to jump to.

**Fig. 8:**
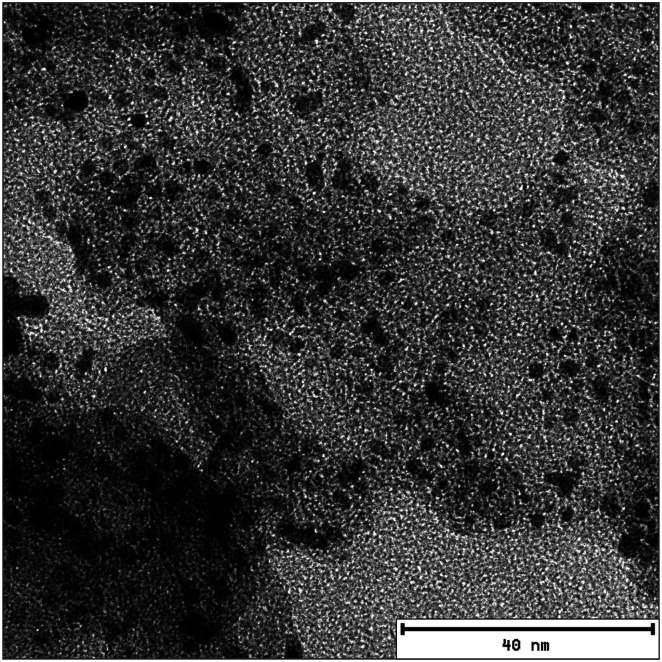
TEM image of the catalyst layer. The Pt particles are clearly visible on the carbon support. The average distance between Pt particles measured on the image is ∼5 ± 2 nm.

In summary, the trap model describes well the proton mobility in the electrode layers and allows to get insight into the trapping of the PA by the Pt catalyst (visible in the TEM image in Fig. 8), and also gives access to the amount of PA which is not affected by trapping.

## 4. Conclusions

The proton dynamics of the phosphoric acid in the catalytic layer of gas diffusion electrodes has been studied using quasielastic neutron scattering. We have compared the dynamics in the catalytic layers with high and low doping levels. Three models based on random jump diffusion have been used to describe the proton diffusion: random jump diffusion, jump diffusion with distribution of the diffusion constant and a trap model. It has been shown that the proton diffusion in the catalytic layer with high doping level can be reasonably described with the jump diffusion model while this approach does not provide consistent results for the sample with low doping level. This indicates that the diffusion in the catalytic layer is more complicated than the diffusion of free PA. Applying a model where the diffusion coefficients are distributed according to a lognormal distribution as described in eqn (4) leads to a broad width of the distribution function and does not provide a realistic physical description of the proton diffusion, especially for the low doping level. Finally, the trap model successfully describes the QENS spectra for both samples with low and high doping levels. The parameters which characterize the traps, namely trapping time and the mean squared distance between the traps, are independent of the doping level and defined by the structure of the catalytic layer. The catalytic layer doped with 100 μl of PA contains additionally 50% of free PA which leads to an extra contribution in the spectra. At the same time, the fraction of the free PA in the catalytic layer with low doping level is almost negligible. Thus, the protons are trapped in the electrode at the surface of the Pt catalyst particles; the influence of the traps on the proton diffusion and the loss of mobility is well described by the jump diffusion model with traps for all temperatures and length scales probed with QENS, which will help in our opinion to assess the influence of Pt catalyst morphology and PA doping levels on possible losses of conductivity in the electrode layer.

The estimated proton conductivity of the free PA obtained from the trap model is in good agreement with reported values. The lower proton conductivity in the catalytic layers can be explained by the trapping mechanism.

## Conflicts of interest

There are no conflicts of interest to declare.

## Supplementary Material

## References

[cit1] Wainright J. S., Wang J. T., Weng D., Savinell R. F., Litt M. (1995). J. Electrochem. Soc..

[cit2] Page K. A. and Rowe B. W., in Polymers for energy storage and delivery: polyelectrolytes for batteries and fuel cells, ed. K.A. Page, C.L. Soles and J. Runt, American Chemical Society, Washington, DC, 2012, pp. 147–164

[cit3] Wannek C., Lehnert W., Mergel J. (2009). J. Power Sources.

[cit4] Wannek C., Konradi I., Mergel J., Lehnert W. (2009). Int. J. Hydrogen Energy.

[cit5] Carrette L., Friedrich K., Stimming U. (2001). Fuel cells.

[cit6] Lehnert W. , Wannek C. and Zeis R., in Innovations in fuel cell technology, ed. R. Steinberger-Wilckens and W. Lehnert, RSC Publishing, Cambridge, 2010, p. 45

[cit7] Asensio J., Sanchez E., Gomez-Romero P. (2010). Chem. Soc. Rev..

[cit8] Zhang J., Xie Z., Zhang J., Tang Y., Song C., Navessin T., Shi Z., Song D., Wang H., Wilkinson D., Liu Z., Holdcroft S. (2006). J. Power Sources.

[cit9] Dippel T., Kreuer K. D., Lassegues J. C., Rodriguez D. (1993). Solid State Ionics.

[cit10] Lüke L., Janßen H., Kvesić M., Lehnert W., Stolten D. (2012). Int. J. Hydrogen Energy.

[cit11] Jalani N. H., Ramani M., Ohlsson K., Buelte S., Pacifico G., Pollard R., Staudt R., Datta R. (2006). J. Power Sources.

[cit12] Lobato J., Canizares P., Rodrigo M. A., Linares J. J. (2007). Electrochim. Acta.

[cit13] Tang Y., Zhang J., Song C., Zhang J. (2007). Electrochem. Solid-State Lett..

[cit14] Vilciauskas L., Tuckerman M. E., Bester G., Paddison S. J., Kreuer K.-D. (2012). Nat. Chem..

[cit15] Lassegues J.-C., Cavagnat D. (1992). Phys. B.

[cit16] Frick B., Vilciauskas L., Deen P., Lyonnard S. (2013). Solid State Ionics.

[cit17] Mader J., Xiao L., Schmidt T. J., Benicewicz B. C. (2008). Adv. Polym. Sci..

[cit18] Wippermann K., Wannek C., Oetjen H.-F., Mergel J., Lehnert W. (2010). J. Power Sources.

[cit19] Li O., He R., Jensen J. O., Bjerrum N. (2004). Fuel Cells.

[cit20] Pahari S., Choudhury C. K., Pandey P. R., More M., Venkatnathan A., Roy S. (2012). J. Phys. Chem. B.

[cit21] Vilciauskas L., Tuckerman M. E., Melchior J. P., Bester G., Kreuer K.-D. (2013). Solid State Ionics.

[cit22] Holderer O., Ivanova O., Hopfenmueller B., Zamponi M., Maier W., Majerus A., Lehnert W., Monkenbusch M., Zorn R. (2014). Int. J. Hydrogen Energy.

[cit23] Lehnert W. , Wannek C. and Zeis R. in Innovations in Fuel Cell Technology, ed. R. Steinberger-Wilckens and W. Lehnert, RSC Publishing, Cambridge, 2010, p. 45

[cit24] Babcock E., Szekely N., Konovalova A., Lin Y., Appavou M.-S., Mangiapia G., Revay Z., Stieghorst C., Holderer O., Henkensmeier D., Lehnert W., Carmo M. (2019). J. Membr. Sci..

[cit25] Ivanova O., Lüke W., Majerus A., Krutyeva M., Szekely N. K., Pyckhout-Hintzen W., Appavou M.-S., Monkenbusch M., Zorn R., Lehnert W., Holderer O. (2017). J. Membr. Sci..

[cit26] Embs J. P., Juranyi F., Hempelmann R. (2010). Z. Phys. Chem..

[cit27] Hopfenmüller B., Zorn R., Holderer O., Ivanova O., Lehnert W., Lüke W., Ehlers G., Jalarvo N., Schneider G. J., Monkenbusch M., Richter D. (2018). J. Chem. Phys..

[cit28] Neutron Applications in Materials for Energy, ed. G.J. Kearley and V. K. Peterson, Springer, Cham, 2015

[cit29] Khaneft M., Holderer O., Ivanova O., Lüke W., Kentzinger E., Appavou M. S., Zorn R., Lehnert W. (2016). Fuel Cells.

[cit30] Wuttke J., Budwig A., Drochner M., Kaemmerling H., Kayser F.-J., Kleines H., Ossovyi V., Carlos Pardo L., Prager M., Richter D., Schneider G. J., Schneider H., Staringer S. (2012). Rev. Sci. Instrum..

[cit31] Heinz Maier-Leibnitz Zentrum (2015). Journal of large-scale research facilities.

[cit32] Bee M. , Quasielastic neutron scattering: principles and applications in solid-state chemistry, biology and materials science, Bristol, 1988

[cit33] van Hove L. (1954). Phys. Rev..

[cit34] Singwi K. S., Sjölander A. (1960). Phys. Rev..

[cit35] Richter D., Springer T. (1978). Phys. Rev. B.

[cit36] Kaserer S., Caldwell K. M., Ramaker D. E., Roth C. (2013). J. Phys. Chem. C.

[cit37] Mauro J. C., Yue Y., Ellison A. J., Gupta P. K., Allan D. C. (2009). Proc. Natl. Acad. Sci..

[cit38] Vogel H. (1921). Phys. Z..

[cit39] Fulcher G. S. (1925). J. Am. Ceram. Soc..

[cit40] Tammann G. (1925). J. Soc. Glass Technol..

[cit41] Chung S. H., Bajue S., Greenbaum S. G. (2000). J. Chem. Phys..

[cit42] Chin D. T., Chang H. H. (1989). J. Appl. Electrochem..

[cit43] Korte C. , Conti F., Wackerl J. and Lehnert W., in High Temperature Polymer Electrolyte Membrane Fuel Cells, ed. Q. Li*et al.*, Springer, 2016

